# G-CNV: A GPU-Based Tool for Preparing Data to Detect CNVs with Read-Depth Methods

**DOI:** 10.3389/fbioe.2015.00028

**Published:** 2015-03-10

**Authors:** Andrea Manconi, Emanuele Manca, Marco Moscatelli, Matteo Gnocchi, Alessandro Orro, Giuliano Armano, Luciano Milanesi

**Affiliations:** ^1^Institute for Biomedical Technologies, National Research Council, Milan, Italy; ^2^Department of Electrical and Electronic Engineering, University of Cagliari, Cagliari, Italy

**Keywords:** CNV, GPU, HTS, read-depth, parallel

## Abstract

Copy number variations (CNVs) are the most prevalent types of structural variations (SVs) in the human genome and are involved in a wide range of common human diseases. Different computational methods have been devised to detect this type of SVs and to study how they are implicated in human diseases. Recently, computational methods based on high-throughput sequencing (HTS) are increasingly used. The majority of these methods focus on mapping short-read sequences generated from a donor against a reference genome to detect signatures distinctive of CNVs. In particular, read-depth based methods detect CNVs by analyzing genomic regions with significantly different read-depth from the other ones. The pipeline analysis of these methods consists of four main stages: (i) data preparation, (ii) data normalization, (iii) CNV regions identification, and (iv) copy number estimation. However, available tools do not support most of the operations required at the first two stages of this pipeline. Typically, they start the analysis by building the read-depth signal from pre-processed alignments. Therefore, third-party tools must be used to perform most of the preliminary operations required to build the read-depth signal. These data-intensive operations can be efficiently parallelized on graphics processing units (GPUs). In this article, we present G-CNV, a GPU-based tool devised to perform the common operations required at the first two stages of the analysis pipeline. G-CNV is able to filter low-quality read sequences, to mask low-quality nucleotides, to remove adapter sequences, to remove duplicated read sequences, to map the short-reads, to resolve multiple mapping ambiguities, to build the read-depth signal, and to normalize it. G-CNV can be efficiently used as a third-party tool able to prepare data for the subsequent read-depth signal generation and analysis. Moreover, it can also be integrated in CNV detection tools to generate read-depth signals.

## 1 Introduction

SVs in the human genome can influence phenotype and predispose to or cause diseases (Feuk et al., 2006a,b). Single nucleotide polymorphisms (SNPs) were initially thought to represent the main source of human genomic variation (Sachidanandam et al., 2001). However, following the advances in technologies to analyze genome, it is now acknowledged that different types of SVs contribute to the genetic makeup of an individual. SV is a term generally used to refer different types of genetic variants that alter chromosomal structure as inversions, translocations, insertions, and deletions (Hurles et al., 2008). SVs such as insertions and deletions are also referred as CNVs. CNVs are the most prevalent types of SVs in the human genome and are implicated in a wide range of common human diseases including neurodevelopmental disorders (Merikangas et al., 2009), schizophrenia (Stefansson et al., 2008), and obesity (Bochukova et al., 2009). Studies based on microarray technology demonstrated that as much as 12% of the human genome is variable in copy number (Perry et al., 2006), and this genomic diversity is potentially related to phenotypic variation and to the predisposition to common diseases. Hence, it is essential to have effective tools able to detect CNVs and to study how they are implicated in human diseases.

Hybridization-based microarray approaches as arraycomparative genomic hybridization (a-CGH) and SNP microarrays have been successfully used to identify CNVs (Carter, 2007). The low cost of a-CGH and SNP platforms promoted the use of microarray approaches. However, as pointed out in Alkan et al. (2011), microarrays (i) have limitations in the task of detecting copy number differences, (ii) provide no information on the location of duplicated copies, and (iii) are generally unable to resolve breakpoints at the single-base-pair level. Recently, computational methods for discovering SVs with HTS (Kircher and Kelso, 2010) have also been proposed (Medvedev et al., 2009). These methods can be categorized into alignment-free (i.e., *de novo* assembly) and alignment-based (i.e., paired-end mapping, split read, and read-depth) approaches (Zhao et al., 2013). The former (Iqbal et al., 2012; Nijkamp et al., 2012) focus on reconstruct DNA fragments by assembling overlapping short-reads. CNVs are detected by comparing the assembled contigs to the reference genome. The latter focus on mapping short-read sequences generated from a donor against the reference genome with the aim of detecting signatures that are distinctive of different classes of SVs. Mapping data hide useful information that can be used to detect different SVs. Different methods that analyze different mapping information have been devised.

*Paired-end mapping* (PEM) methods (Chen et al., 2009; Korbel et al., 2009; Sindi et al., 2009; Hormozdiari et al., 2010, 2011; Mills et al., 2011) identify SVs/CNVs by detecting and analyzing paired-end reads generated from a donor that are discordantly mapped against the reference genome. These methods allow to detect different types of SVs (i.e., insertions, deletions, mobile element insertions, inversions, and tandem duplications), but they do not allow to detect insertions larger than the average insert size of the library preparations.

*Split read* (SR) methods (Ye et al., 2009; Abel et al., 2010; Abyzov and Gerstein, 2011; Zhang et al., 2011) are also based on paired-end reads. Unlike PEM methods that analyze discordant mappings, SR methods analyze unmapped or partially mapped reads as they potentially provide accurate breaking points at the single-base-pair level for SVs/CNVs.

**Figure 1:**
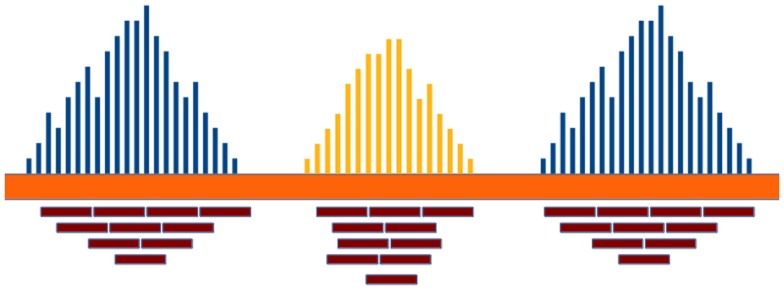
**The RD in a genomic region depends on the copy number of that region and follows a Poisson distribution**. Duplicated and deleted regions are characterized by a RD signal different from that of the other ones.

**Figure 2:**
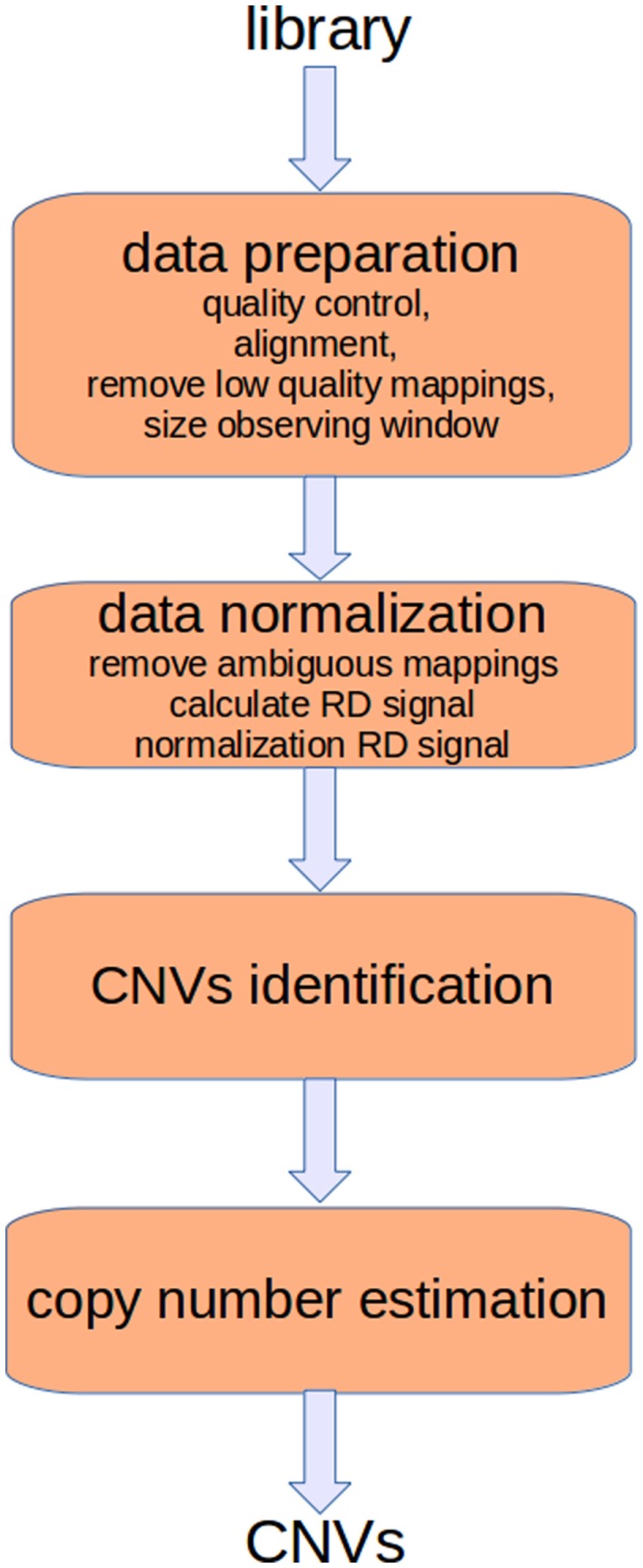
**The analysis pipeline of RD-based methods consists of four main stages**. The first two stages consist of preparatory operations aimed at generating the RD signal. Sequencing produces artifacts that affect the alignment and consequently the RD signal. Different filtering operators can be applied to reduce these errors. Moreover, alignments must be post-processed to remove those of low quality and to resolve ambiguities. Finally, the RD signal is calculated taking into account the bias related with the GC-content.

*Read-depth* (RD) methods (Chiang et al., 2008; Xie and Tammi, 2009; Yoon et al., 2009; Ivakhno et al., 2010; Xi et al., 2010; Abyzov et al., 2011; Miller et al., 2011) are based on the assumption that the RD in a genomic region depends on the copy number of that region. In fact, as the sequencing process is uniform, the number of reads aligning to a region follows a Poisson distribution with mean directly proportional to the size of the region and to the copy number [see Figure 1 and Chiang et al. (2008)]. These methods analyze the RD of a genome sequence through non-overlapping windows, with the aim of detecting those regions that exhibit a RD significantly different from the other ones. A duplicated region will differ from the other ones for a higher number of reads mapping on it, and then for a higher RD. Conversely, a deleted region will differ from the other ones for a lower number of reads mapping on it, and then for a lower RD. Basically, the analysis pipeline implemented in RD methods consists of four fundamental stages (Magi et al., 2012): (i) data preparation; (ii) data normalization; (iii) CNV regions identification; and (iv) copy number estimation (see Figure 2).

Data preparation consists of different tasks aimed at assessing the quality of the read sequences, mapping the reads against the reference genome, removing low mapping quality sequences, and sizing the observing window used to calculate the RD signal. Data normalization is aimed at correcting the effect of two sources of bias that affect the detection of CNVs. In particular, it has been proved that correlation exists between RD and the GC-content (Dohm et al., 2008; Hillier et al., 2008; Harismendy et al., 2009); the RD increases with the GC-content of the underlying genomic region. Moreover, there exists a mappability bias due to the repetitive regions in a genome. A read can be mapped to different positions so that ambiguous mappings must be dealt with. After normalization, RD data are analyzed to detect the boundaries of regions characterized by changed copy number. Finally, DNA copy number of each region within breakpoints is estimated.

The first two stages of the analysis pipeline consist of common operations, whereas the last two consist of specific operations for each method. However, it should be pointed out that available tools do not implement most of the operations required at the first and second stage. Typically, these tools start the analysis by building the RD signal from the post-processed alignments. All preparatory operations must be performed by the researchers using third-party tools. Moreover, other tools as ReadDepth (Miller et al., 2011) require annotation files with information about the GC-content that are pre-computed only for some reference genome builds. Only some tools provide limited functionalities to pre-process alignments. For instance, RDXplorer (Yoon et al., 2009) and CNV-seq (Xie and Tammi, 2009) use the samtools (Li et al., 2009a) to remove low-quality mappings and to select the best hit location for each mapped read sequence, respectively.

Most of these operations are data-intensive and can be parallelized to be efficiently run on GPUs to save computing time. GPUs are hardware accelerators that are increasingly used to deal with computationally intensive algorithms. Recently, GPU-based solutions have been proposed to cope with different bioinformatics problems (e.g., Manavski and Valle, 2008; Liu et al., 2010; Shi et al., 2010; Yung et al., 2011; Manconi et al., 2014a,b; Zhao and Chu, 2014).

In this work, we present GPU-copy number variation (G-CNV), a GPU-based tool aimed at performing the preparatory operations required at the first two stages of the analysis pipeline for RD-based methods. G-CNV can be used to (i) filter low-quality sequences, (ii) mask low-quality nucleotides, (iii) remove adapter sequences, (iv) remove duplicated reads, (v) map read sequences, (vi) remove ambiguous mappings, (vii) build the RD signal, and (viii) normalize it. Apart the task of removing adapter sequences, all the other tasks are implemented on GPU. G-CNV can be used as a third-party tool to prepare the input for available RD-based detection tools or can be integrated in other tools to efficiently build the RD signal.

G-CNV is freely available for non-commercial use. The current release can be downloaded at the following address http://www.itb.cnr.it/web/bioinformatics/gcnv

## 2 Materials and Methods

Data preparation and data normalization are crucial operations to properly detect CNVs. It is widely known that sequencing is a process subject to errors. These errors can affect the alignments; hence both the RD signal and the accuracy of the identified CNVs can be affected as well. G-CNV implements filtering operators aimed at correcting some errors related to the sequencing process. In particular, G-CNV is able to analyze the read sequences to filter those read sequences that do not satisfy a quality constraint, to mask low-quality nucleotides with an a*N*y symbol, to remove adapter sequences, and to remove duplicated read sequences. G-CNV uses *cutadapt* (Martin, 2011) to remove adapter sequences. As for the alignment, G-CNV uses the GPU-based short-read mapping tool SOAP3-dp (Luo et al., 2013). Low-quality alignments are filtered out, while ambiguous mappings can be treated according to different strategies. To build the RD signal, G-CNV builds a RD signal according to a fixed-size observing window. Then, this raw RD signal is corrected according to the GC-content of the observed windows.

In this section, we first give a short introduction to GPUs. Then, we present the strategies adopted to cope with the tasks implemented by G-CNV. Finally, we briefly recall the hardware and software equipment required to use G-CNV.

### 2.1 GPU

**Figure 3:**
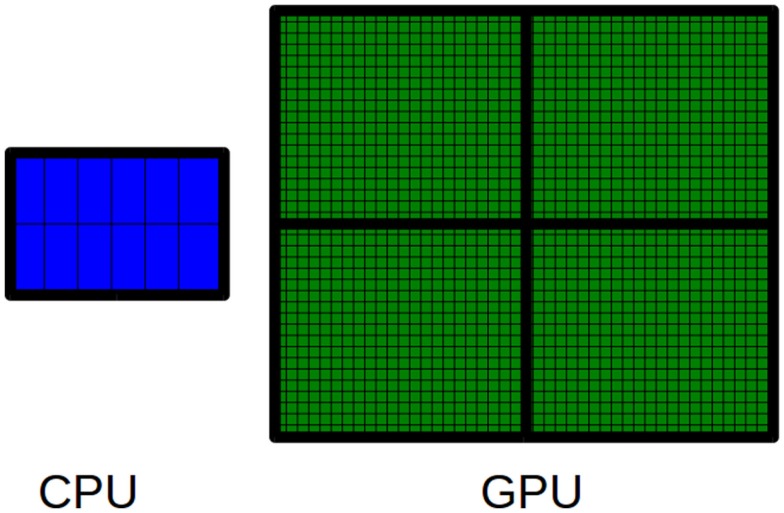
**The main difference between CPUs and GPUs is related to the number of available cores**. A CPU is a multi-core processor that consists of a few cores. Conversely, a GPU is a many-core processor that consists of thousands of available cores.

GPUs are hardware accelerators that are increasingly used to deal with computationally intensive algorithms. From an architectural perspective, GPUs are very different from traditional CPUs. Indeed, the latter are devices composed of few cores with lots of cache memory able to handle a few software threads at a time. Conversely, the former are devices equipped with hundreds of cores able to handle thousands of threads simultaneously, so that a very high level of parallelism can be reached (see Figure 3). Apart from the high level of parallelism, there may be other advantages to use the GPU technology. In particular, the low cost for accessing to the GPUs (if compared with the cost to equip a laboratory with a CPU-cluster) is promoting the technology. Moreover, GPUs are inherently more energy efficient than other ways of computation because they are optimized for throughput and performance per watt and not absolute performance. The main disadvantage of adopting the GPU technology is related with the effort required to code algorithms. GPUs can run certain algorithms very faster than CPUs. However, gaining this speed-up can require a notably effort to properly code the algorithms for GPU. Algorithms must be coded to reflect the GPU architecture. To do this can mean to dive into the code and make significant changes to several parts of the algorithm. For the sake of completeness, it should be pointed out that depending on the algorithm may be more advantageous to parallelize it on CPUs rather than on GPUs. Due to their significantly different architectures, CPUs and GPUs can be suited to address different tasks. Therefore, both CPU and GPU parallelism offer particular advantages for particular problems.

**Figure 4:**
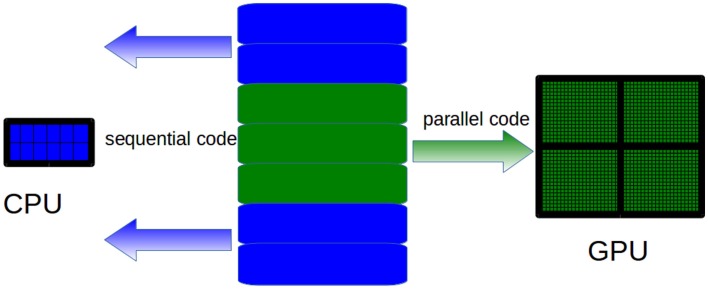
**CPUs are more effective than GPUs to run sequential code**. Therefore, only the computationally intensive parts of the algorithms must be run on the GPU, whereas sequential parts must be run on CPUs.

The GPU computing model uses both a CPU and a GPU in a heterogeneous co-processing computing model. As a CPU is more effective than a GPU for serial processing, it is used to run the sequential parts of an algorithm, whereas computationally intensive parts are accelerated by the GPU (see Figure 4). It should be pointed out that the task of the CPU is not limited to just control the GPU execution. Hybrid CPU/GPU parallelization can also be implemented depending on the algorithm. As for GPU programing, Compute Unified Device Architecture (CUDA) (Nvidia, 2007) and Open Computing Language (OpenCL) (Munshi et al., 2009) offer two different interfaces for programing GPU. OpenCL is an open standard that can be used to program CPUs, GPUs, and other devices from different vendors. CUDA is specific to NVIDIA GPUs.

**Figure 5:**
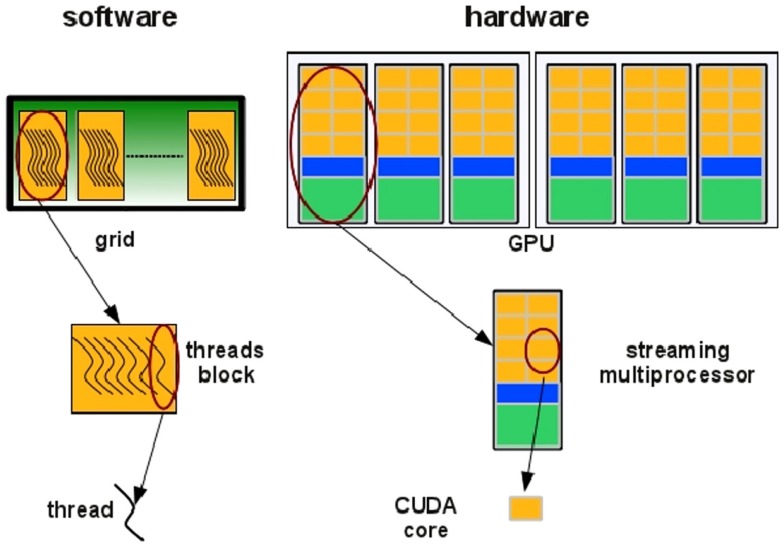
**Threads are grouped in blocks in a grid**. Each thread has a private memory and runs in parallel with the others in the same block (Manconi et al., 2014a).

In the NVIDIA GPU-based architecture, parallelization is obtained through the execution of tasks in a number of stream processors or CUDA cores. Cores are grouped in multiprocessors that execute in parallel. A CUDA core executes a floating point or integer instruction per clock cycle for a thread and all cores in a streaming multiprocessor execute in a Single Instruction Multiple Thread (SIMT) fashion. All cores in the same group execute the same instruction at the same time. SIMT can be considered an extension of the Single Instruction Multiple Data (SIMD) paradigm: basically, the SIMD paradigm describes how instructions are executed whereas the SIMT paradigm also describes how threads are executed. The code is executed in groups of threads called warps. Device memory access takes a very long time due to the very long memory latency. The parallel programing model of the CUDA architecture provides a set of API that allows programmers to access the underlying hardware infrastructure and to exploit the fine-grained and coarse-grained parallelism of data and tasks. Summarizing, the CUDA execution model (see Figure 5) can be described as follow: the GPU creates an instance of the kernel program that is made of a set of threads grouped in blocks in a grid. Each thread has a unique ID within its block and a private memory and registers, and runs in parallel with others threads of the same block. All threads in a block execute concurrently and cooperatively by sharing memory and exchanging data. A block, identified by a unique ID within the block grid, can execute the same kernel program with different data that are read/written from a global shared memory. Each block in the grid is assigned to a streaming multiprocessor in a cyclical manner.

### 2.2 Quality control

The sequencing technology has been notably improved. Modern sequencers are able to generate hundreds of millions of reads in a single run and the sequencing cost is rapidly decreasing. Despite this improvement, sequencing data are affected by artifacts of different nature that may strongly influence the results of the research. Hence, the ability to assess the quality of read sequences and to properly filter them are major factors that determine the success of a sequencing project. In particular, as for RD methods, both low quality and duplicated read sequences affect the RD signal and consequently the identification of CNV regions.

Different tools have been proposed for quality control of sequencing data such as NGS QC Toolkit (Patel and Jain, 2012), HTQC (Yang et al., 2013), FASTX-Toolkit^1^, FASTQC^2^, and Picard^3^. Most of these tools support both Illumina and 454 platforms, while only some of them support CPU parallelization. It should be pointed out that the artifacts generated during the sequencing process and the massive amount of generated reads make quality control tasks difficult and computationally intensive. The massive parallelization that can be provided by GPUs can be used to deal with these computational tasks. Starting from this assumption, we integrated G-CNV with GPU-based operators to filter low-quality sequences, to mask low-quality nucleotides, and to detect and remove duplicated read sequences. Only the removing of adapter sequences has not yet been implemented on GPU. Currently, these operators are specialized for short-read sequences generated with Illumina platforms.

#### 2.2.1 Filtering low-quality sequences

FASTQ files report quality values for each sequence. Basically, G-CNV parses these files to identify low-quality nucleotides. Nucleotides are classified as of low quality if their quality value is lower than a user-defined threshold. FASTQ files represent quality values using an ASCII encoding. Different encodings are used depending on the Illumina platform. Illumina 1.0 format encodes quality scores from −5 to 62 using ASCII 59 to 126. From Illumina 1.3 and before Illumina 1.8, quality scores ranges from 0 to 62 and are encoded using ASCII 64–126. Starting in Illumina 1.8, quality scores range from 0 to 93 and are encoded using ASCII 33–126.

G-CNV performs filtering in three steps. The first step is performed on CPU, whereas the last two steps are massively parallelized on a single GPU. As for the first step, G-CNV analyses the FASTQ files to detect the Illumina format. Then, the quality values of sequences are decoded according to the detected Illumina format. Finally, G-CNV removes those read sequences that exhibit a percentage of low-quality nucleotides that exceed a user-defined threshold. As a final result, a new FASTQ file is created with the filtered sequences so that the original FASTQ file is preserved.

#### 2.2.2 Masking low-quality nucleotides

G-CNV can also be used to mask low-quality nucleotides. Similarly that for the filtering of low-quality sequences, G-CNV performs masking in three steps. The first step is performed on CPU and it is aimed at detecting the Illumina format. Conversely, the last two steps are massively parallelized on a single GPU and are aimed at decoding the quality values sequences according to the Illumina format, and at masking with an a*N*y symbol those nucleotides with a quality score lower than a user-defined threshold. Then, a new FASTQ file is created with the masked nucleotides.

#### 2.2.3 Removing adapter sequences

In the current release, G-CNV uses *cutadapt* to remove adapter sequences. *Cutadapt* can be used to look for adapter sequences in reads generated with Illumina, 454, and SOLiD HTS machines. Basically, *cutadapt* is able to look for multiple adapters in the 5′ and 3′ ends according to different constraints (e.g., mismatches, indels, minimum overlap between the read and adapter). It can be used to trim or discard reads in which an adapter occurs. Moreover, it allows to automatically discard those reads that after the trimming are shorter than a given user-defined length. All features of *cutadapt* were wrapped in G-CNV.

It should be pointed out that the current release of *cutadapt* is not parallelized. In order to speed up the removing of the adapters, G-CNV splits the original FASTQ files in chunks and runs in parallel an instance of *cutadapt* on each of these chunks. Finally, the output files provided by each instance of *cutadapt* are merged together in a new FASTQ file.

#### 2.2.4 Removing duplicated read sequences

Duplicate reads are one of the most problematic artifacts. These artifacts are generated during the PCR amplification. Ideally, duplicates should have identical nucleotide sequences. However, due to the sequencing errors, they could be nearly identical (Gomez-Alvarez et al., 2009). Alignment-based [e.g., NGS QC Toolkit, SEAL (Pireddu et al., 2011), and Picard MarkDuplicates] and alignment-free [e.g., FastUniq (Xu et al., 2012), Fulcrum (Burriesci et al., 2012), CD-HIT (Li and Godzik, 2006; Fu et al., 2012)] methods have been proposed in the literature to remove duplicated read sequences. Basically, alignment-based methods start from the assumption that duplicated reads will be mapped into a reference genome in the same position. Therefore, in these methods, read sequences are aligned against a reference genome and those reads with identical alignment positions are classified as duplicates. It should be pointed out that the final result is affected by both the alignment constraints and the accuracy of the aligner. In alignment-free methods, read sequences are compared among them according to a similarity measure. The reads with a similarity score lower than a given threshold are classified as duplicated.

**Figure 6:**
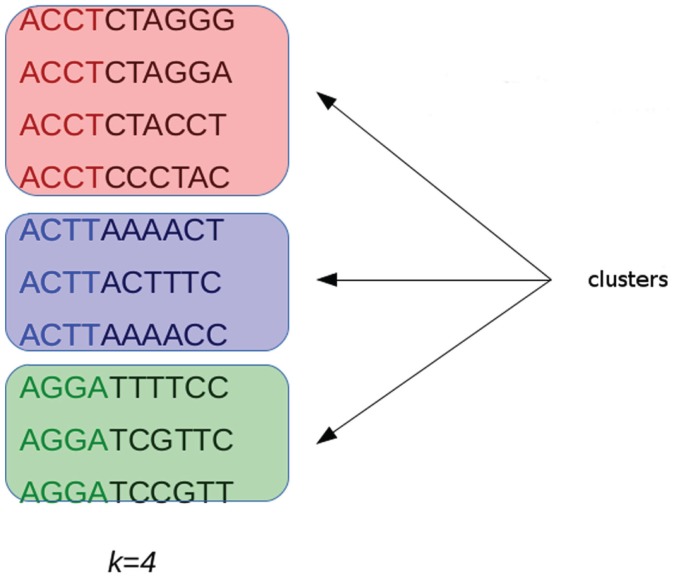
**Short-reads with an identical prefix (of fixed length *k*) are clustered together as potential duplicated sequences**. This approach takes into account the error rates of Illumina platforms. Analysis performed on short-reads generated with these sequencing platforms highlighted that the number of wrong bases increases with the base position.

**Figure 7:**
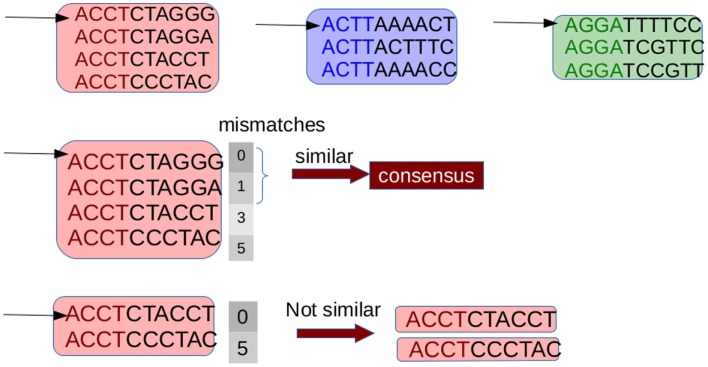
**Suffixes of sequences in a cluster are compared to identify the duplicates**. The first read is taken as a seed and its suffix is compared with those of the other ones. Sequences with a number of mismatches lower than a given threshold are considered duplicates of the seed. These sequences are removed from the cluster and are represented with a consensus sequences. Then the process is repeated until the cluster is empty or consists of a single sequence.

G-CNV implements an alignment-free method to remove duplicated read sequences from single-end libraries. Like other tools, it implements a prefix–suffix comparison approach. The algorithm has been devised taking into account the per-base error rates of Illumina platforms. Analysis of short-read datasets obtained with Illumina highlighted a very low rate of indel errors (<0.01%) while the number of occurrences of wrong bases increases with the base position (Dohm et al., 2008). Therefore, G-CNV does not take into account indels and considers as potentially duplicated read sequences those with an identical prefix. Potential duplicated sequences are clustered together (see Figure 6), and for each cluster G-CNV compares the suffixes of its sequences. The first sequence of a cluster is taken as a seed and its suffix is compared with those of the other sequences in that cluster. Those sequences identical or very similar to the seed are considered duplicated. Duplicated sequences will be condensed in a new sequence and will be removed from the cluster (see Figure 7). Then, the process is iterated for the remaining sequences in the cluster (if any), until the cluster is empty or contains only a read sequence.

**Figure 8:**
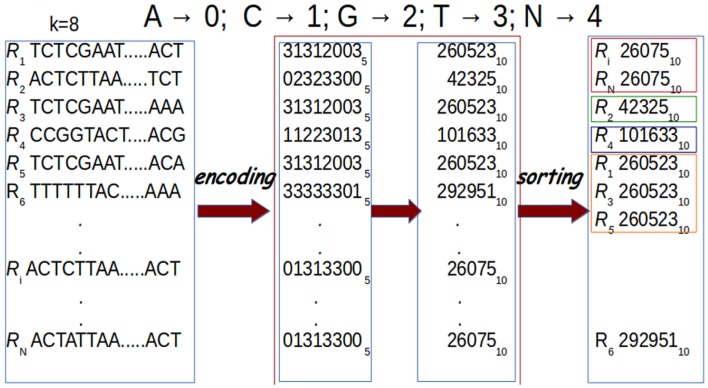
**Prefixes are subject to a dual encoding**. As for the first encoding, each nucleotide in a prefix is represented with a numerical value from 0 to 4 (*A* → 0, *C* → 1, *G* → 2, *T* → 3, *N* → 4). Then, these numerical representations are encoded using base-10. Finally, sorting is performed for clustering. In the figure, prefixes of length *k* = *8* are represented.

In G-CNV, clustering is performed sorting the prefixes of the read sequences. Sorting is performed on a GPU with our CUDA-Quicksort^4^^,^^5^. Experimental results show that CUDA-Quicksort is faster than other available GPU-based implementations of the quicksort. In particular, it results be up to 4 times faster than GPU-Quicksort of Cederman and Tsigas (2008) and up to 3 times faster than the NVIDIA CDP-Quicksort (CUDA toolkit 6.0). As CUDA-Quicksort sorts numerical values, the prefixes must necessarily be subject to a numerical encoding. We devised the encoding with the aim to maximize the length of the prefixes that can be compared. In doing this, read sequence prefixes are subject to a dual numerical encoding. Initially, we encoded the prefixes using a base-5 encoding by replacing each nucleotide with a numerical value ranging from 0 to 4 (i.e., *A* → 0, *C* → 1, *G* → 2, *T* → 3, *N* → 4). Using CUDA-Quicksort to sort items represented with *64 bit unsigned long long int* data type, prefixes of up to 19 nucleotides can be sorted. A longer prefix will exceed the limit for this type of data. However, it is possible to exceed this constraint using a different numerical base to represent the prefixes. In particular, using the base-10, it is possible to represent a number consisting of 27 digits with a *64 bit unsigned long long int* (see Figure 8). Therefore, G-CNV applies this second encoding to maximize the length of the prefixes used for clustering.

**Figure 9:**
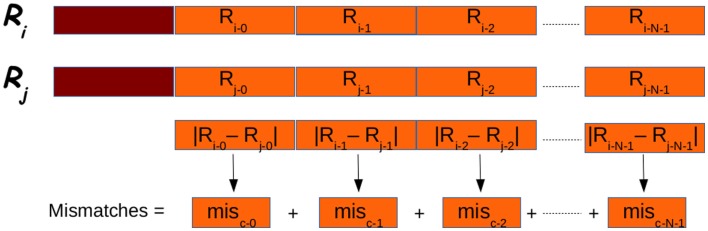
**Suffixes (in orange in the figure) are analyzed in chunks**. Each chunk is subject to the dual encoding used for prefixes (in red in the figure). The overall number of mismatches if obtained summing the partial number of mismatches obtained for each chunk.

After that the reads have been clustered G-CNV compares their suffixes. This step requires a base-per-base comparison of the nucleotides of the seed read sequence with those of the other reads in a cluster. This approach can require a very high number of base–base comparisons. Let *N* be the length of the suffixes, and let *m* be the allowed number of mismatches. In the best case, *m* comparisons must be performed to classify two sequences as not duplicated. In the worst case, *N*–*m* comparisons must be performed to classify two sequences as duplicated. Apart from the high number of comparisons required, this approach is not adapted to be efficiently implemented on GPUs. As GPUs adopt the SIMT paradigm, each thread in a block must perform the same operation on different data. Then, G-CNV implements a different comparison method. Suffixes are split into fixed length chunks. Subsequence of each chunk is subjected to the same dual numerical encoding used to represent the prefixes for clustering. Then for each cluster, the numerical difference between the *i-*th chunk of the seed and the related chunk of the other suffixes in a cluster is calculated (see Figure 9). The order of magnitude of the difference provides information about the position of the leftmost different nucleotides. Then, the subsequences are cut corresponding to the mismatch position. The rightmost parts of the mismatch position are maintained and the process is re-iterated.

### 2.3 Mapping

It is widely known that mapping of short-read sequences is computationally onerous. Several tools have been devised to deal with short-read mappings. Without claiming to be exhaustive, let us cite some of the most popular solutions, i.e., MAQ (Li et al., 2008), RMAP (Smith et al., 2008, 2009), Bowtie (Langmead et al., 2009), BWA (Li and Durbin, 2009), CloudBurst (Schatz, 2009), SOAP2 (Li et al., 2009b), and SHRiMP (Rumble et al., 2009; David et al., 2011). A comparative study aimed at assessing the accuracy and the runtime performance of different cutting-edge next-generation sequencing read alignment tools highlighted that among all, SOAP2 was the one that showed the higher accuracy (Ruffalo et al., 2011). Exhaustive review of the tools cited above can be found in Bao et al. (2011).

In general, the mentioned solutions exploit some heuristics to find a good compromise between accuracy and running time. Recently, the GPU-based short-read mapping tools Barracuda (Klus et al., 2012), CUSHAW (Liu et al., 2012b), SOAP3 (Liu et al., 2012a), and SOAP3-dp have been successfully proposed to the scientific community. In particular, SOAP3-dp aligns the read sequences in two steps. As for the first step, it looks for ungapped alignments with up to four mismatches without using heuristics. As for the second step, it uses the dynamic programing to look for gapped alignments. Compared with BWA, Bowtie2 (Langmead and Salzberg, 2012), SeqAlto (Mu et al., 2012), GEM (Marco-Sola et al., 2012), and the previously mentioned GPU-based aligners, SOAP3-dp is two to tens of times faster, while maintaining the highest sensitivity and lowest false discovery rate on Illumina reads with different lengths.

Starting from the previous analysis, we decided to use SOAP3-dp to support read mapping in G-CNV. G-CNV allows to set different parameters of SOAP3-dp that can be useful to properly generate alignments for RD methods. Apart from the constraints on the allowed mismatches, G-CNV allows to set SOAP3-dp parameters able to filter out alignments that are not of interest for the specific RD method. In particular, as different methods presented in the literature filter alignments using different quality mapping scores, G-CNV allows to set a quality mapping threshold on the alignments that must be reported. To set these constraints, G-CNV needs to be able to access the SOAP3-dp files to change the initialization file. Moreover, a short-read may be uniquely aligned or can be aligned to multiple positions onto a genome. Multiple mappings can be related to the alignment constraints or to the nature of the sequenced read. A read sequence can be aligned to multiple positions, as it has been sequenced from repetitive regions or regions of segmental duplication (Abyzov et al., 2011). In the former case, alignments are characterized by different alignment scores, whereas in the latter case, they are expected to have equal or very similar scores. A common approach to take into account multiple mappings is to randomly select a best alignment. G-CNV allows to report only unique best alignments or a random best alignment.

### 2.4 RD signal

The RD signal depends on the size of the observing window. As methods proposed in the literature suggest different approaches to estimate the window size, G-CNV does not impose it. In G-CNV, the window size is a parameter that must be set by the user.

G-CNV builds the RD signal in two steps. Initially, G-CNV analyses the genome sequences to build a GC-content signal according to the fixed window size. A GC-signal for each genome sequence will be built. Then, G-CNV splits the mapping for each chromosome sequences, identifies the window where the mappings fall, and calculates a raw RD signal. By default, the window related to each alignment is identified considering the center of the read. Finally, G-CNV corrects the RD signal with the same approach proposed in Yoon et al. (2009) that adjust the RD by using the observed deviation of RD for a given GC percentage according to the following equation:

(1) $$RD_{w_{i}}^{\prime }=\frac{\bar{\mathrm{RD}}}{{\bar{\mathrm{RD}}}_{GC_{w_{i}}}}\cdot RD_{w_{i}}$$

where *RD_wi_* is the RD for the *i*-th window to be corrected, $\bar{\mathrm{RD}}$ is the average RD signal, ${\bar{\mathrm{RD}}}_{GC_{w_{i}}}$ is the average RD signal calculated on the windows with the GC-content found in the *i*-th window, and ${\mathrm{RD}}_{w_{i}}^{\prime }$ is the corrected RD for the *i*-th window.

### 2.5 Hardware and software requirements

G-CNV has been designed to work with NVIDIA GPU cards based on the most recent Kepler architecture. G-CNV works on Linux-based systems equipped with CUDA (release ≥6.0). We tested it on the NVIDIA Kepler architecture-based k20c card. Experiments have been carried out using the last release of *soap3-dp* (rel. 2.3.177) and of *cutadapt* (rel. 1.7.1).

## 3 Results

We performed different experiments aimed at assessing the performance of G-CNV. In particular, we assessed its performance when used to filter low-quality sequences, to mask low-quality nucleotides, to remove adapter sequences, to remove duplicated reads, and to calculate the RD signal. Since G-CNV performs the alignments running *SOAP3-dp*, we deemed not relevant to assess the performance of G-CNV in this task. We invite the readers to refer the *SOAP3-dp* manuscript for an in-depth analysis of the performance of the aligner. Similarly, as G-CNV uses the well-known tool *cutadapt* to remove adapter sequences, we did not perform tests aimed at assessing its reliability in this task. However, we performed experiments aimed at assessing the benefits of the parallelization of *cutadapt* provided with G-CNV.

Experiments have been carried out on both synthetic and real-life libraries. Synthetic reads have been used to assess and compare with other tools the reliability of G-CNV, whereas real-life data to assess and compare its performance in terms of both computing time and memory consumption.

Synthetic reads have been generated from the build 37.3 of the human genome using the *Sherman* simulator^6^. *Sherman* has been devised to simulate HTS datasets for both bisulfite sequencing and standard experiments. To mimic real data, it generates synthetic data using an error rate curve that follows an exponential decay model. We used *Sherman* to generate a single-end synthetic library consisting of 1 millions of 100 bp reads. Library has been generated simulating a sequencing error of 2% and contaminating the reads with the Illumina single-end adapter 1 (i.e., ACACTCTTTCCCTACACGACGCTGTTCCATCT). The contamination has been simulated with a normal distribution of fragment sizes. Moreover, since *Sherman* generates identical quality scores for all reads, we modified them to generate a 3% of low-quality nucleotides (PHRED value ≤20) and a 9% of low-quality sequences. In the following of the manuscript, we will refer to this dataset as the *S1* library.

Since *Sherman* does not permit to control the percentage of duplicates, we modified the simulated reads in *S1* to generate a new synthetic library (*S2*) consisting of 30% of duplicated sequences. Read sequences have been duplicated simulating a sequencing error of 2%. The library *S1* has been used to assess the reliability of G-CNV in the task of filtering low-quality sequences and masking low-quality nucleotides, whereas *S2* has been used to assess the reliability of G-CNV in the task of removing duplicated sequences.

As for real-life data, experiments have been performed on different libraries generated with Illumina platforms: (i) SRR001220 consisting of 3.3 millions of 94 bp reads; (ii) SRR001205 consisting of 9.7 millions of 47 bp reads; (iii) SRR005720 consisting of 26.2 millions of 36 bp reads; and (iv) SRR921889 consisting of 50 millions of 100 bp reads (see Table 1).

**Table 1:** **Real-life datasets**.

Dataset	Library layout	Reads (M)	Read size (bp)	Organism	Instrument
SRR001220	Single	3.3	94	*Homo sapiens*	Illumina Genome Analyzer II
SRR001205	Single	9.7	47	*Homo sapiens*	Illumina Genome Analyzer II
SRR005720	Paired	26.2	36	*Homo sapiens*	Illumina Genome Analyzer
SRR921889	Single	50.0	100	*Mus musculus*	Illumina HiSeq 2000

*The first column reports the name of the dataset. The second column reports the library layout. The third and fourth columns report the size of the dataset and the length of the reads, respectively. Organism and sequencing instrument are reported in column fifth and sixth*.

Moreover, with the aim to simulate a CNV pre-processing detection analysis, we simulated two high coverage (30x) whole genome sequencing experiments. The first experiment have been simulated generating 37 synthetic libraries consisting of 25 millions of 100 bp reads, and the second generating 9 synthetic libraries consisting of 100 millions of 100 bp reads. All libraries have been generated according to the same constraints used to generate *S1*. In the following of the manuscript, we will refer to these datasets as *HCS1* and *HCS2*.

In the following of this section, we describe the different experiments and present results. Finally, we briefly resume the hardware and software configuration used for experiments.

### 3.1 Filtering low-quality sequences

To assess G-CNV in the task of filtering low-quality read sequences, we compared its performance with those of FASTX-Toolkit and NGS QC Toolkit. Experiments have been performed setting parameters with the aim to filter those sequences with a percentage of low-quality (PHRED score <20) bases >10% (see Table 2).

**Table 2:** **Tools settings used to filter low-quality sequences**.

Tool	
G-CNV	−mf 20 −pf 90
FASTX-Toolkit	-Q33 -q 20 -p 90
NGS QC Toolkit^a^	N A -l 90 -s 20
NGS QC Toolkit^b^	N A -l 90 -s 20 -c 12

*Both FASTX-Toolkit and NGS QC Toolkit consist of different commands. As for FASTX-Toolkit, experiments have been performed using the fastq_quality_filter command, whereas the IlluQC_PRLL has been used for NGS QC Toolkit. The table reports the settings used to run NGS QC Toolkit without exploiting parallelization (NGS QC Toolkit ^a^) and parallelized on 12 CPU cores (NGS QC Toolkit ^b^)*.

A first experiment has been performed on the *S1* synthetic library aimed at assessing and comparing the reliability of G-CNV with the other tools. As expected, all tools have been able to filter all low-quality sequences. The same experiment has been performed on the real-life libraries aimed at assessing the performance of G-CNV in terms of both computing time and memory consumption. It should be pointed out that FASTX-Toolkit does not support parallelization whereas in NGS QC Toolkit parallelization has been implemented in multiprocessing and multithreaded ways. Multiprocessing parallelization was implemented to process multiple files in parallel whereas multithreading parallelization to process in parallel a single file. The FASTQ file is split into chunks, processed in parallel, and results are merged at the end. With the aim to provide an in-depth comparison among all tools and to assess as NGS QC Toolkit can scale increasing the CPU cores, we initially run the experiments without using parallelization, then experiments have been performed parallelizing the computation on 12 CPU cores.

It should be pointed out that FASTX-Toolkit does not provide support for paired-end libraries. Therefore, it has not been possible to test it with the *SRR005720* dataset. Experimental results show that G-CNV is most effective than the other tools in terms of computing time. Table 3 reports computing time and peak of memory required by G-CNV, FASTX-Toolkit, and NGS QC Toolkit to analyze the different datasets. G-CNV has been 12.4x/7.8x/NA/21.4x faster than FASTX-Toolkit and 24x/21x/26.5x/28.3x faster than NGS QC Toolkit parallelized on 12 CPU cores to filter the read sequences of the SRR001220/SRR001205/SRR005720/SRR921889 dataset. Obviously, the performance of G-CNV improves notably when compared with those of NGS QC Toolkit executed without parallelization. In this case, G-CNV has been 154x/120x/125x/175x faster than NGS QC Toolkit to analyze the SRR001220/SRR001 205/SRR005720/SRR921889 dataset.

**Table 3:** **Performance evaluation to filter low-quality sequences**.

Tool	Dataset	Filtered seq. (%)	Time	Memory
G-CNV	SRR001220	95.3	5 s	0.9 GB
	SRR001205	98.3	11 s	1.4 GB
	SRR005720	74.7	48 s	4.5 GB
	SRR921889	7.9	1 min 10 s	10.5 GB
FASTX-Toolkit	SRR001220	95.3	1 min 2 s	256 KB
	SRR001205	98.3	1 min 19 s	256 KB
	SRR005720	–	–	–
	SRR921889	7.9	17 min 10 s	256 KB
NGS QC Toolkit^a^	SRR001220	95.3	12 min 52 s	0.21 GB
	SRR001205	98.3	22 min	0.18 GB
	SRR005720	74.7	1 h 40 min	0.26 GB
	SRR921889	7.9	3 h 25 min	0.22 GB
NGS QC Toolkit^b^	SRR001220	95.3	2 min	1.4 GB
	SRR001205	98.3	3 min 52 s	1.4 GB
	SRR005720	74.7	21 min	1.3 GB
	SRR921889	7.9	33 min	1.9 GB

*The first and the second column of the table report the tool and the analyzed library, respectively. The third column the percentage of filtered reads. Column fourth reports the computing time required to analyze the different libraries. The fifth column the peak of memory required to perform the analysis*.

For the sake of completeness, it should be pointed out that NGS QC Toolkit automatically also generates statistics for quality check. Therefore, the computing time reported from NGS QC Toolkit takes into account also the time required to perform these operations.

As for the memory consumption, FASTX-Toolkit is undoubtedly the most effective tool. Conversely, G-CNV requires more memory than the other tools. Its performance is only comparable with those of NGS QC Toolkit executed in parallel for the *SRR001220* and *SRR001205* datasets. Experimental results show that the memory required by G-CNV increases with the size of the analyzed library. This is mainly due to the fact that to massively parallelize the computation G-CNV loads into the memory as many as possible read sequences to maximize the occupancy of the grid of the GPU.

Finally, we used G-CNV to filter the low-quality sequences of the *HCS1* and *HCS2* datasets. Filtering has been performed in ~20 min for the *HCS1* and in ~34 min for *HCS2*. As for the memory consumption, G-CNV required 5.7 GB to analyze *HCS1* and 20.5 GB for *HCS2*.

### 3.2 Masking low-quality nucleotides

The performance of G-CNV in the task of masking low-quality nucleotides have only been compared with those of FASTX-Toolkit. NGS QC Toolkit does not provide support for this operator. G-CNV and FASTX-Toolkit have been run to mask with a a*N*y symbol the nucleotides with a PHRED quality score <20 (see Table 4). Experiments performed on the *S1* synthetic library shown that both tools have been able to mask all low-quality sequences. Experiments performed on real-life libraries show that G-CNV outperforms notably FASTX-Toolkit in terms of computing time. Results reported in Table 5 show that G-CNV has been 12x/6.8x/5x/13.8x faster than FASTX-Toolkit to analyze the SRR001220/SRR001205/SRR005720/SRR921889 dataset. As previously highlighted, FASTX-Toolkit does not support paired-end reads. However, as for the task of masking low-quality nucleotides, it can be separately used on both the forward and the reverse read sequences. Then, as for the *SRR005720* dataset Table 5 reports the overall computing time required by FASTX-Toolkit to analyze both files.

**Table 4:** **Tools settings used to mask low-quality nucleotides**.

Tool	
G-CNV	-m 20
FASTX-Toolkit	-Q33 -q 20 -r N

*As for FASTX-Toolkit experiments have been performed using the fastq_quality_masker command*.

**Table 5:** **Performance evaluation to mask low-quality nucleotides**.

Tool	Dataset	Masked nucl. (%)	Time	Memory
G-CNV	SRR001220	24.2	5 s	0.94 GB
	SRR001205	43.6	10 s	1.38 GB
	SRR005720	21.8	52 s	3.88 GB
	SRR921889	3	1 min 15 s	12 GB
FASTX-Toolkit	SRR001220	24.2	1 min	256 KB
	SRR001205	43.6	1 min 8 s	256 KB
	SRR005720	21.8	4 min 22 s	256 KB
	SRR921889	3	17 min 20 s	256 KB

*The first and the second column of the table report the tool and the analyzed library, respectively. The third column the percentage of masked nucleotides. Column fourth reports the computing time required to analyze the different libraries. The fifth column the peak of memory required to perform the analysis*.

As for the high coverage-simulated sequencing experiments, G-CNV masked the low-quality nucleotides of *HCS1* in ~23 min using 7 GB of memory, whereas required ~39 min and 21.9 GB of memory for *HCS2*.

### 3.3 Removing adapter sequences

As for the task of removing adapter sequences, G-CNV has been compared with both FASTX-Toolkit and NGS QC Toolkit. To assess the advantages of the implemented parallelization of *cutadapt*, we initially performed experiments running G-CNV without exploiting the parallelization, subsequently parallelizing the computation on 12 CPU cores. Tool settings used to perform these experiments are reported in Table 6.

**Table 6:** **Tools settings used to remove adapter sequences**.

Tool	
G-CNV^a^	–ca-a ACACTCTTTCCCTACACGACGCTGTTCCATCT
G-CNV^b^	–ca-a ACACTCTTTCCCTACACGACGCTGTTCCATCT –ca-t 12
FASTX-Toolkit	–Q33 -a ACACTCTTTCCCTACACGACGCTGTTCCATCT
NGS QC Toolkit^a^	≪ ADAPTER FILE ≫ A
NGS QC Toolkit^b^	≪ ADAPTER FILE ≫ A -c 12

*As for FASTX-Toolkit experiments have been performed using the fastx_clipper command, whereas the IlluQC_PRLL has been used for NGS QC Toolkit. In the table have been reported the settings used to run G-CNV^a^ and NGS QC Toolkit^a^ without exploiting the parallelization and in multithreading way (G-CNV^b^ and NGS QC Toolkit^b^). The table shows the settings used to remove the Illumina Single-End Adapter 1. As for the SRR005720 dataset, settings have been modified to remove the Illumina paired-end adapters*.

Table 7 reports results obtained analyzing the real-life libraries. Results show that the performance of G-CNV improves notably with parallelization. With parallelization G-CNV has been 6.7x/6.4x/23.4x/2.8x faster to remove the adapter sequences from the SRR001220/SRR001205/SRR005720/SRR921889 dataset. Moreover, G-CNV parallelized on 12 CPU cores resulted to be 18.2x/11x/–/9.4x faster than FASTX-Toolkit and 11.8x/7.3x/58.3x/6.3x NGS QC Toolkit used exploiting the parallelization to remove the adapters from the SRR001220/SRR001205/SRR005720/SRR921 889 dataset. Obviously, also for this task the performance of G-CNV improves when compared with NGS QC Toolkit used without parallelization. In this case, G-CNV resulted be 48x/40x/173x/38x faster than NGS QC Toolkit to analyze the SRR001220/SRR001205/SRR005720/SRR921889 dataset. As for the memory consumption, FASTX-Toolkit provides better performance than the other tools. However, G-CNV outperforms NGS QC Toolkit. As FASTX-Toolkit does not support paired-end libraries, it has not been used to analyze the *SRR005720* dataset.

**Table 7:** **Performance evaluation to remove adapter sequences**.

Tool	Dataset	Time	Memory
G-CNV^a^	SRR001220	1 min 14 s	17 MB
	SRR001205	2 min 46 s	21 MB
	SRR005720	8 min 12 s	26 MB
	SRR921889	17 min 11 s	20 MB
G-CNV^b^	SRR001220	11 s	0.4 GB
	SRR001205	26 s	0.46 GB
	SRR005720	21 s	0.33 GB
	SRR921889	6 min 10 s	0.84 GB
FASTX-Toolkit	SRR001220	3 min 21 s	516 KB
	SRR001205	4 min 47 s	516 KB
	SRR005720	–	–
	SRR921889	57 min 40 s	516 KB
NGS QC Toolkit^a^	SRR001220	8 min 52 s	217 MB
	SRR001205	17 min 30 s	189 MB
	SRR005720	1 h 48 min	269 MB
	SRR921889	3 h 55 min	226 MB
NGS QC Toolkit^b^	SRR001220	2 min 10 s	1.6 GB
	SRR001205	3 min 10 s	1.3 GB
	SRR005720	20 min 24 s	1.13 GB
	SRR921889	39 min	1.6 GB

*The first and the second column of the table report the tool and the analyzed library, respectively. Column third reports the computing time required to analyze the different libraries. The fourth column the peak of memory required to perform the analysis*.

Finally, when used to remove adapters from the *HCS1*, G-CNV required ~50 min and 250 MB of memory, whereas it required ~3 h 20 min and 920 MB for *HCS2*.

### 3.4 Removing duplicated read sequences

To assess the performance of G-CNV in the task of removing duplicated sequences, we compared its performance with those of Fulcrum. G-CNV implements a very similar algorithm to that implemented in Fulcrum. In particular, similarly to our tool, Fulcrum clusters together the reads with a similar prefix and looks for duplicates in the same cluster.

Table 8 reports the main parameters that have been used for the experiments. Experiments on the synthetic *S2* library have been performed clustering reads according to a prefix length of 25 bp and looking for identical sequences (i.e., 0 mismatches) and nearly identical sequences with up to 1 mismatch. Results reported in Table 9 show that both tools have been able to identify the synthetic duplicate sequences. It should be pointed out that *S2* has been built avoiding to generate mismatches among the duplicated sequences in their first 25 bp. As for tests on real-life data, we performed experiments on the larger *SRR921889* dataset. Experiments have been aimed at assessing the performance of G-CNV to remove duplicated sequences according to different constraints. In particular, we performed the experiments on both G-CNV and Fulcrum to cluster sequences according to a prefix size of 10 and 25 bp and to look for duplicated sequences with up to 1 and up to 3 mismatches. Experimental results are reported in Table 10. For each experiment were reported the percentage of removed sequences, the computing time and the peak of memory required for the analysis. Results show that both tools remove a similar percentage of duplicated sequences. However, as for the computing time, G-CNV outperforms Fulcrum in all experiments. It should be pointed out that Fulcrum automatically parallelize the computation on all available CPU cores. Therefore, the computing times reported in the table have been obtained running Fulcrum parallelized on 12 CPU cores. Results show that the computing time required by G-CNV depends on both the number of allowed mismatches and the prefix size. The number of sequences that will be classified as duplicated increases with the number of allowed mismatches. Therefore, increasing this value may involve a lower number of sequences comparison. Moreover, the size of a cluster depends on the prefix length. Typically, the size of the clusters increases as the prefix length decreases involving more sequences comparison.

**Table 8:** **Tools settings used to remove duplicated sequences**.

Tool	
G-CNV	-D ≪*mis* ≫-p ≪*pref* ≫
Fulcrum	-b ≪*pref* ≫-s -t s -c ≪*mis* ≫

*We performed different experiments with different values for both the prefix length and the allowed mismatches. Specific values for the prefixes ≪pref ≫ and the allowed mismatches ≪mis ≫ are reported in the tables of the results*.

**Table 9:** **Performance evaluation to remove duplicated sequences from the synthetic S2 library**.

Tool	Dataset	Mismatches	Percentage removed
G-CNV	S2	0	0
	S2	1	30.1
Fulcrum	S2	0	0
	S2	1	30.6

*The first column reports the tool. The second column reports the dataset. Columns third and forth report the allowed mismatches and the percentage of removed duplicated sequences*.

**Table 10:** **Performance evaluation to remove duplicated sequences from the real life dataset SRR921889**.

Tool	Prefix	Mismatches	Percentage removed	Time	Memory
G-CNV	10	1	11.2	2 h	17.3 GB
	10	3	11.5	1 h 50 min	17.3 GB
	25	1	11.9	16 min	17.3 GB
	25	3	12.1	8 min	17.3 GB
Fulcrum	10	1	11.3	4 h 01 min	1.6 GB
	10	3	11.4	3 h 23 min	1.6 GB
	25	1	11.6	1 h 24 min	1.6 GB
	25	3	11.9	1 h 33 min	1.6 GB

*The first column reports the tool. The second column reports the length of the prefixes used for clustering. Column third reports the allowed mismatches. The fourth column reports percentage of removed sequences. Columns fifth and sixth report the computing time and the memory consumption, respectively*.

For the sake of completeness, G-CNV performed the clustering step in ~2 s for both length of the prefixes, whereas Fulcrum required 13 min to cluster the reads according to a prefix of 10 bp and 56 min to cluster the reads according to prefix length of 25 bp. However, it should be pointed out that G-CNV can not be used to cluster reads with a prefix longer than 27 bp. Moreover, the clustering phase implemented by G-CNV requires that all prefixes will be loaded into the memory of the GPU device. This implies a constraint on the size of the analyzed library, which depends on the memory of the GPU. As for the memory consumption, G-CNV undoubtedly requires more memory than Fulcrum. Also in this case, the high memory consumption is due to the need of maximize the occupancy of the grid of the GPU.

Finally, we performed different experiments on the *HCS1* dataset. Experiments have been performed to cluster the reads according to a prefix length of 15 and 27 bp and to look for duplicated with up to 1 and to 3 mismatches. Results are reported in Table 11.

**Table 11:** **Performance evaluation to remove duplicated sequences from the synthetic HCS1 dataset**.

Mismatches	Prefix	Time	Memory
1	15	12 h 7 min	8.8 GB
	27	5 h 33 min	6.6 GB
3	15	3 h 20 min	8.7 GB
	27	1 h 30 min	5.7 GB

*The first column reports the allowed mismatches. The second column reports the length of the prefix used to cluster the reads. Columns third and fourth report the computing time and memory consumption, respectively*.

### 3.5 Generating the RD signal

As there are no other specialized tools to generate the RD signal, we cannot assess and compare the performance of G-CNV with other tools. However, we used the *FastQC* tool^7^ to assess the reliability of G-CNV in the task of calculating the GC-content that is used to normalize the RD signal. FastQC is a tool that provides some quality control checks on HTS data. In particular, it is able to calculate the distribution of the per-sequence GC-content of the analyzed read sequences.

As G-CNV calculates the GC-content of each observed window in the genome sequences, we generated a synthetic library using as reads the subsequences observed with a window of 100 bp along the MT chromosome of the human genome (build 37.3). Then, we used FastQC to analyze the GC-content of these sequences and compared the results with those generated by G-CNV. Both tools provided the same distribution of the GC-content. It should be pointed out that it was not possible to compare the results with those of FASTX-Toolkit and NGS QC Toolkit as both determine only the per-base GC-content. We did not compare the time required by G-CNV with that required by *FastQC* as it automatically performs several quality checks.

Moreover, to assess the performance of G-CNV to generate a RD signal, we simulated an alignment SAM file on the human genome (build 37.3). The alignment has been simulated by assuming a sequencing experiment on the genome with coverage 30x. We did not simulate sequencing and alignment errors. The SAM file was generated by assuming an ideal aligner able to map the reads uniquely and without errors. In fact, these errors do not affect the computing time to generate the RD signal; they affect the detection of CNVs. However, as for this experiment, we have been mainly interested to assess the computing time of G-CNV in the task of generating the RD signal. G-CNV generated the RD signal with an observing window of length 100 in less than 1 h 56 min and required 10.4 GB of memory. As for the memory used by G-CNV, it depends on the number of alignments in the analyzed genome sequence. G-CNV generates the RD signal analyzing separately the genome sequences. To maximize the parallelization, as many as possible alignments on the analyzed genome sequence are loaded into the GPU.

### 3.6 Hardware and software configuration

Experiments described hereinafter have been carried out on a 12 cores Intel Xeon CPU E5-2667 2.90 GHz with 128 GB of RAM and an NVIDIA Kepler architecture-based Tesla k20c card with 0.71 GHz clock rate and equipped with 4.8 GB of global memory.

## 4 Discussion

Different RD-based methods and tools have been proposed in the literature to identify CNVs. Typically, these tools do not support most of the preparatory operations for RD analysis. Therefore, a specific analysis pipeline must be built with different third-party tools. G-CNV allows to build the analysis pipeline required to process short-read libraries for RD analysis according to different constraints. However, in our opinion, the added value of G-CNV is the fact that almost all operations are performed on GPUs. In fact, these are data-intensive operations that may require an enormous computing power. GPUs are increasingly used to deal with computational intensive problems. The low cost for accessing the technology and their very high computing power is facilitating the GPUs success. Experimental results show that G-CNV is able to efficiently run the supported operations. However, it should be pointed out that the current release of G-CNV still has some limitations and/or constraints. In particular, as for removing duplicates, there are two main limitations of our algorithm. As for the former, the current release of G-CNV supports removal of duplicates only for single-end reads. As for the latter, there exists a constraint on the clustering phase. Sorting requires that all prefixes will be loaded into the memory of the GPU device. This implies a constraint on the size of the analyzed library, which depends on the memory of the GPU. With a GPU card equipped with 4.8 GB of global memory, libraries of up to 220 M reads can be analyzed. A solution to overcome this constraint is to parallelize the sorting on multiple GPU devices. We are currently working to adapt CUDA-Quicksort to run on multiple GPUs. Although CUDA-Quicksort resulted be the fastest GPU-based implementation of the quicksort algorithm, the Thrust Radix Sort is currently the fastest GPU-based sorting algorithm. However, as for clustering, we adapted and used our CUDA-Quicksort as it has been designed to be easily modified to scale on multiple GPUs. Moreover, we deem that the overall performance of G-CNV can be improved by implementing the trimming of the adapters on GPUs.

## Author Contributions

Conceived the tool: AM. Conceived and designed the experiments: AM, LM. Performed the experiments: AM, MG, EM, GA. Analyzed the data: AM, AO, EM, GA, MM, MG, LM. Wrote the manuscript: AM. Revised the manuscript: AM, GA, LM. Wrote the program: AM. Generated the synthetic data: AM. Coordinated the project: LM.

## Conflict of Interest Statement

The authors declare that the research was conducted in the absence of any commercial or financial relationships that could be construed as a potential conflict of interest.

## Footnotes

^1^http://hannonlab.cshl.edu/fastx_toolkit/

^2^http://www.bioinformatics.babraham.ac.uk/projects/fastqc/

^3^http://broadinstitute.github.io/picard/

^4^http://sourceforge.net/projects/cuda-quicksort/

^5^Submitted to Concurrency and Computation: Practice and Experience: manuscript CPE-14-0292 entitled “CUDA-Quicksort: an improved GPU-based implementation of Quicksort”

^6^http://www.bioinformatics.babraham.ac.uk/projects/sherman/

^7^http://www.bioinformatics.babraham.ac.uk/projects/fastqc/
